# Supplementary material for: Synthesis and Determination of Thermotropic Liquid Crystalline Behavior of Cinnamaldehyde-Based Molecules with Two Schiff Base Linking Units
Source: Molecules. 2020 Aug 20;25(17):3780. doi: 10.3390/molecules25173780 (PMC7504561; doi:10.3390/molecules25173780)
Supplement: Supplementary file 1 [file molecules-25-03780-s001.pdf]

Supplementary Materials

Table S1. FTIR data of *p*-hydroxybenzaldehyde, **1a**, and **1b**.

**Compounds**	**Linkage (cm^{-1})**					
	**O-H stretch**	**C$_{sp3}$-H stretch**	**H-CO, aldehydic**	**C=O stretch**	**C=C stretch**	**C-O stretch**
***p*-hydroxybenzaldehyde**	3200-3400	-	-	1700	1604	1164
**1a**	-	2928, 2734	2700	1687	1599	1157
**1b**	-	2922, 2733	2701	1691	1600	1157

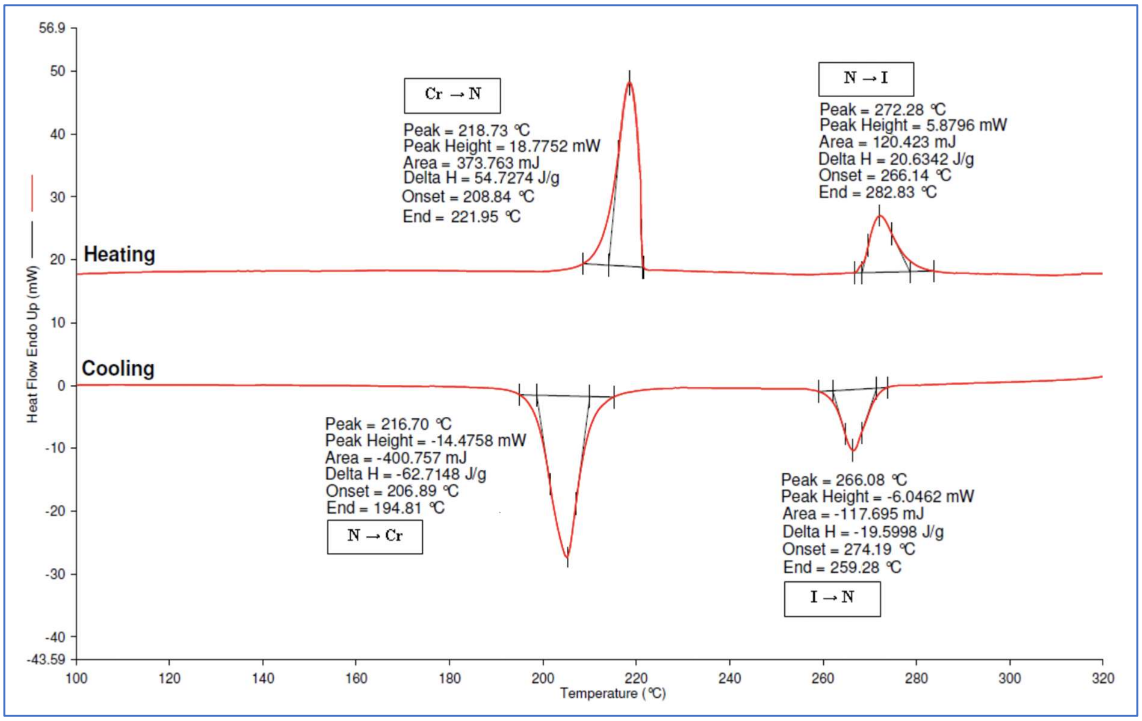

Figure S1. DSC thermogram of compound **2**.

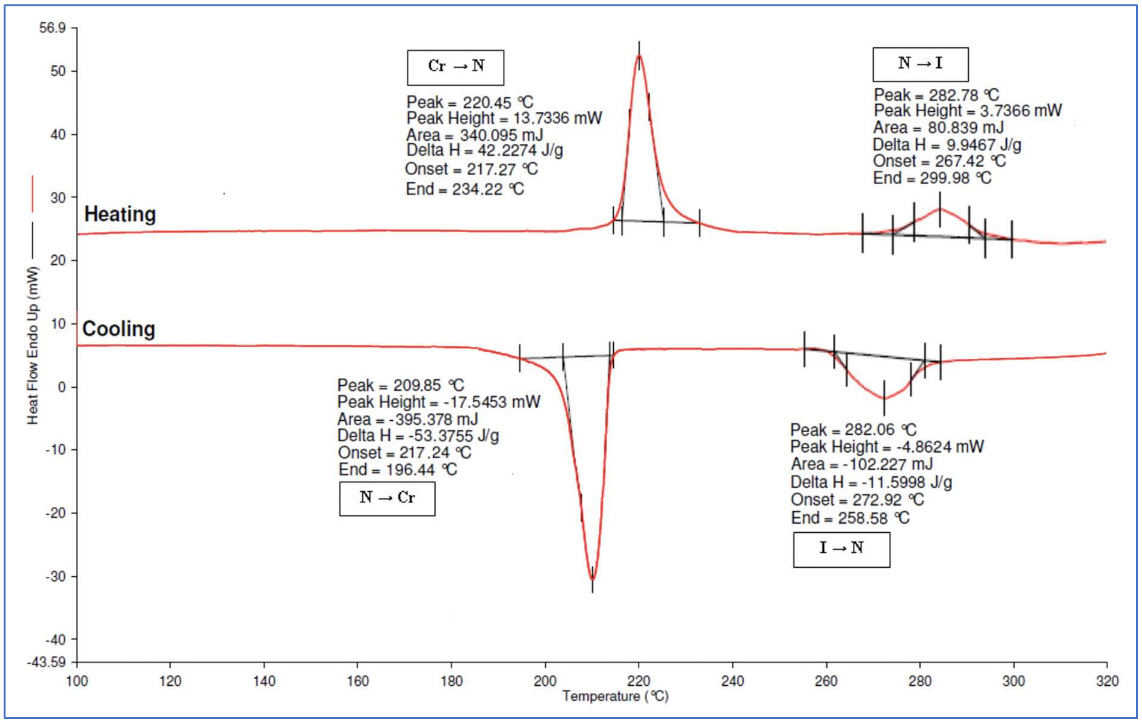

Figure S2. DSC thermogram of compound **3**.

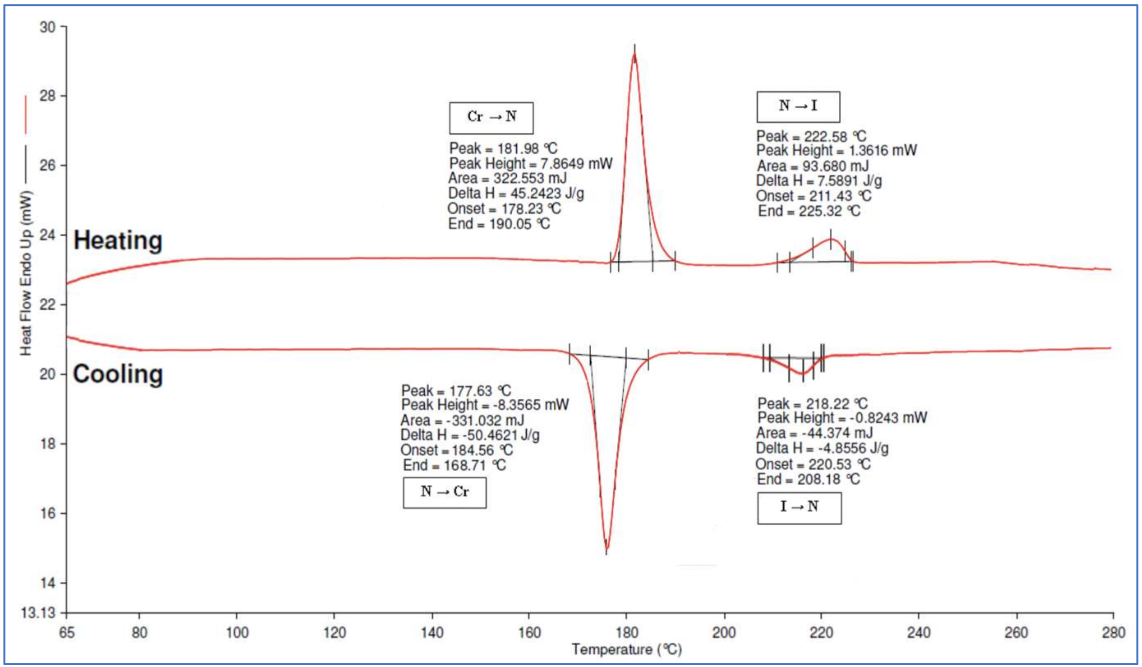

Figure S3. DSC thermogram of compound **7**.

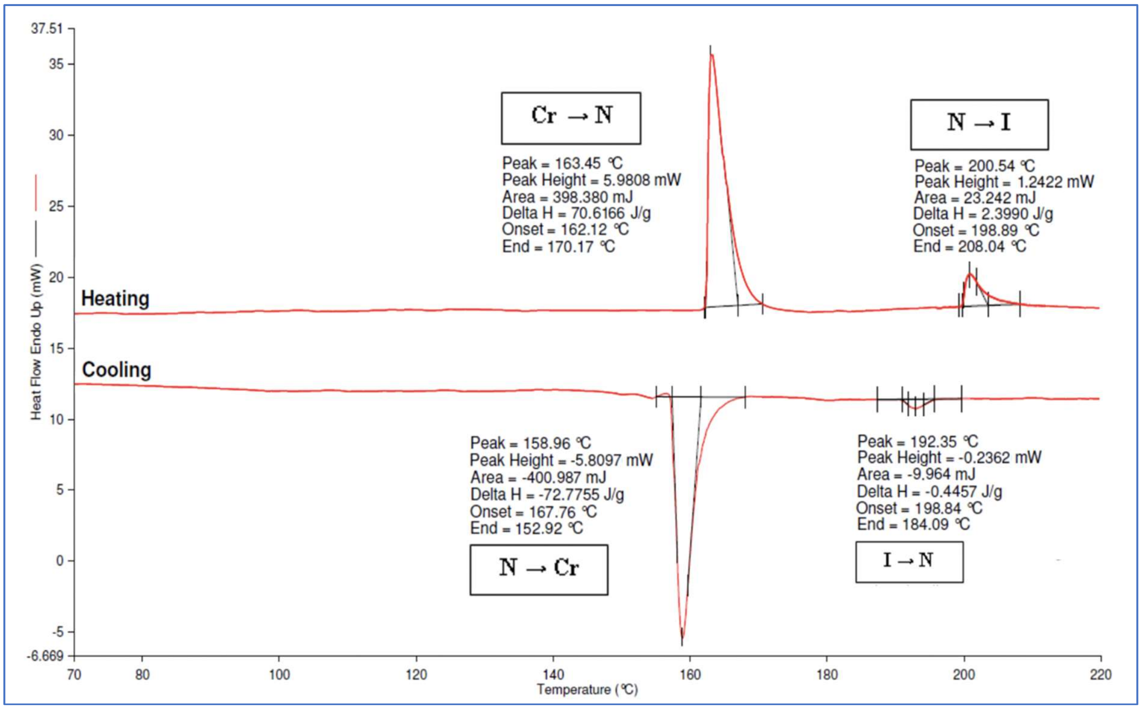

Figure S4. DSC thermogram of compound **9**.
